# Numeracy Skills, Cognitive Reserve, and Psychological Well-Being: What Relationship in Late Adult Lifespan?

**DOI:** 10.3390/bs10110176

**Published:** 2020-11-22

**Authors:** Maria Chiara Fastame, Silvia Melis

**Affiliations:** Department of Pedagogy, Psychology, Philosophy, University of Cagliari, Via Is Mirrionis 1, 09123 Cagliari, Italy; silvia.melis@live.it

**Keywords:** numeracy, psychological well-being, mental calculation, number comprehension, aging, cognitive reserve, mental health, metacognition, physical health

## Abstract

Background: The capacity of understanding and manipulating numerical stimuli (i.e., numeracy) can impact decision making. This investigation was conducted to examine whether number comprehension and mental calculation predict hedonic (i.e., Scale of Positive and Negative Experience, SPANE) and eudaimonic (i.e., Flourishing Scale) well-being in late adulthood, and whether cognitive reserve (i.e., education, time spent for gardening, and time spent for leisure activities) and non-verbal reasoning predict numeracy skills of old adults. Additionally, the effect of age on numeracy was examined, controlling for the effect of education and cognitive efficiency. Methods: One hundred and fifty-eight (i.e., 65–94 years old) community-dwellers completed a battery of tools assessing numeracy, cognitive and metacognitive efficiency, and psychological well-being. Results: Number comprehension, metacognition, time spent for leisure, and perceived physical health accounted for 23% of the variance in the SPANE condition, whereas metacognition, perceived physical health, time for leisure, and education explained 15% of the variance in the Flourishing condition. Moreover, cognitive reserve assessed in terms of vocabulary and education predicted mental calculation. Finally, aging significantly impacted the mental calculation performance of older participants. Conclusions: These findings suggest that numeracy skills can selectively impact the mental health and daily life of older adults.

## 1. Introduction

The occurrence of the global rise of the older population—that in 2017 doubled compared to 1980 and that is expected to reach approximately 2.1 billion in 2050, that is, 1 in 6 people in the world will be at least 65 years old—significantly impacts social policies for the promotion of different facets of life quality in late adulthood, such as physical and mental health [1].

A relatively recent trend of research highlighted that the capacity of understanding and manipulating numerical stimuli (i.e., numeracy or numerical acuity) can influence decision making, that, in turn, can be crucial for the solution of problems in the daily life of older people, such as for health-related decision making [2]. In this regard, Golbeck et al. [3] defined health numeracy as “the degree to which individuals have the capacity to access, process, interpret, communicate, and act on numerical, quantitative, graphical, biostatistical, and probabilistic health information needed to make effective health decisions” (p. 375). A growing body of studies documented that the ability to understand and process quantitative information can impact the medical decisions of older individuals, including the degree to which one is prone to follow medical advice (i.e., therapeutic adherence). Indeed, to adhere to the therapeutic plans, the person has to evaluate the magnitude of the potential risks and advantages related to the adherence to the medication regimens or screenings, he/she has to understand the frequency and quantity of the medicines to be taken, and the patient has to comprehend the probability (i.e., percentage of success) of surviving in case of successful therapeutic compliance [2,4]. Thus, numerical skills can be very relevant in late lifespan, when the probability of occurrence of chronic morbidity increases (e.g., cardiovascular problems, increased risk of type 2 diabetes), and the need for understanding number-related information (e.g., tables explaining the effect of medicine) is crucial for appropriate medical decision making and the promotion of health-related outcomes [5,6]. In this regard, recent research suggests that older people—especially older women—with low numeracy acuity and less efficient executive functions (i.e., higher-level cognitive processes encompassing planning, inhibition of irrelevant information, cognitive flexibility for the selection of the goal to pursue, decision making, problem-solving, abstraction, and concept formation [7]), mainly those with mild signs of cognitive impairment are vulnerable to the risk of lower number comprehension (e.g., probability, frequency, proportions). The direct consequences of the lower numerical acuity of women are the reduced use and search of health information, which, in turn, negatively impact the access to preventive health services and adversely affect medical compliance and the occurrence of healthy behaviors (e.g., balanced diet). In short, the direct consequence of the reduced numeracy skills in late adulthood is the significant risk of hospitalization and mortality [2,8,9,10]. Consistently, a recent trend of research also documented that older people exhibiting higher financial literacy (i.e., the capacity of accessing, understanding, and using numerical information and financial concepts for the maintenance of life quality through economic outcomes such as saving and investment behaviors [11]) are at a lower risk of hospitalization, since they make more appropriate health-related decisions and invest their money in conducting healthier behaviors [12].

Further studies documented that different socio-demographic (e.g., age, gender), and cognitive factors (e.g., visuo-spatial abstract reasoning, executive functions, general cognitive efficiency) are significantly related to the efficiency of the numeracy skills in late lifespan [13,14,15,16,17]. For instance, in recent a cross-national European study, Barbosa et al. [18] documented that older women were 1.5-fold more subjected to exhibit numeracy deficits than men. Additionally, it should be noticed the paucity of studies concerning the impact of cognitive reserve (i.e., a latent construct that can be assessed through the assessment of its dimensions, such as educational attainment, vocabulary, engagement with in leisure activities [19]) on the numeracy acuity of the older people. To our knowledge, the recent empirical evidence is quite limited and controversial. Indeed, according to Semenza, et al. [20], cognitive reserve (i.e., assessed in terms of formal schooling and past working activity) does not predict written and mental calculation of older individuals, whereas time spent for leisure activities accounted for the ability of the older individuals to manipulate numerical stimuli to solve daily life problems (e.g., using money for shopping). Following this, Mulas, Ruiu, and Fastame [21] documented that more educated older individuals performed better on some written multiplication and addition problems, respectively. Consistently, Barbosa et al. [18] showed that the educational attainment of older individuals was strongly associated with their ability to perform mental subtractions. Furthermore, the authors showed that cognitive reserve (i.e., assessed in terms of vocabulary knowledge and educational attainment) mediated the relationship between non-verbal reasoning and numerical computation. In contrast, according to Arcara et al. [22] cognitive reserve does not predict the ability to perform the mental and written calculation. Finally, according to Delazer et al. [2], less educated older people (i.e., especially women and those belonging to ethnic minorities) living alone and exhibiting lower numerical acuity, display less trust in their cognitive and numerical competencies, therefore they risk less correct health-oriented decision making. Thus, a direct consequence of low numerical acuity in late adulthood is the consequent frequent onset of adverse mental health outcomes (e.g., depressive symptomatology) and the greatest occurrence of hospitalization [23].

Furthermore, at present, there is controversial evidence about the impact of numerical acuity on the perceived mental health outcomes of older adults [24]. Thus far, to our knowledge, the limited literature is focused on the relationship between numeracy skills and depressive symptoms in late adulthood, and the outcomes are inconsistent and do not allow us to track any conclusion. Indeed, Bennett et al. [8] documented that low health literacy (i.e., that includes numeracy skills) is associated with increased depressive signs in late adulthood. Additionally, a recent pilot study documented that cognitively older people who reported to have been financially exploited (e.g., victims of fraud or scam) and therefore lost some financial incomes displayed milder levels of depression and sleep difficulties than the non-financially exploited group [25]. Similarly, in a study conducted with 23–83 years old participants, it was found that lower perceived numeracy was related to lower perceived physical health and higher self-reported negative mood status [26]. Nonetheless, these findings were not replicated by Agarwal et al. [27]. To our knowledge, evidence about the relationship between numerical acuity and perceived hedonic (i.e., a way of feeling well and happy, avoiding distress and unpleasant emotional states, and promoting pleasure and satisfaction with life) and eudaimonic (i.e., a motivation for human action-oriented to the promotion of a “flourishing life, that is, a life full of purpose, meaning and values”) well-being in late lifespan [7] is lacking. Indeed, we are aware of only one recent study [28], conducted with community-dwelling Italian older individuals, documenting that self-assessed depressive signs were predicted by number comprehension, self-reported confidence about one’s mental efficiency (i.e., an index of metacognitive efficiency), perceived physical health, and time spent for gardening. Bearing in mind the relevance of self-reported mental health indexes as a subjective indicator of successful aging, e.g., [29,30], from our viewpoint it is crucial to explore whether numeracy skills impact self-perceived hedonic and eudaimonic well-being in late adulthood.

This investigation was primarily carried out to examine whether numeracy skills contribute to cognitive reserve (i.e., assessed in terms of time spent for gardening and time spent for outdoor socially-oriented leisure activities), metacognition, and perceived physical health in predicting hedonic and eudaimonic well-being in late adulthood. Moreover, a further goal was to examine whether some cognitive reserve measures (i.e., assessed in terms of time spent for leisure activities, vocabulary, and education) mediate on the relationship between non-verbal reasoning and number comprehension and mental calculation, respectively. Additionally, the effect of age-related factors on numeracy acuity was examined, controlling for the effect of formal education and global cognitive efficiency.

Considering the limited and fragmentary available evidence, it was hypothesized that:

(1) Metacognition, perceived physical health, and time spent on leisure activities (i.e., a measure of the cognitive reserve) were expected to predict perceived mental well-being indexes [31,32];
(2) vocabulary (i.e., a measure of the cognitive reserve) and abstract reasoning were expected to be related to numerical skills in late adulthood [14,16,22];
(3) education (i.e., a measure of the cognitive reserve) was expected to mediate on the relationship between non-verbal reasoning and numeracy skills of the older individuals [18,21];
(4) age-related factors were expected to impact number comprehension and mental calculation in late adulthood [2].

Finally, no specific hypotheses about the role of the numerical skills in predicting hedonic and eudaimonic well-being measures of older individuals were proposed, since relevant prior research is lacking.

## 2. Materials and Methods

### 2.1. Participants

One hundred and fifty-eight community-dwelling older participants (65–94 years old, mean age = 76.2 years, SD = 6.3) were recruited in some rural villages located in Sardinia (Italy). The sample was composed of 95 females and 63 males. Participants were equally distributed in the young-old (i.e., 65–74-year-old) and very-old (i.e., > 74 years of age) groups, respectively (*χ*^2^ = 0.911, df = 1, *p* = 0.34).

Educational attainment was dichotomized as low (i.e., ≤ 8 years) and high (i.e., >8 years). As expected from previous studies conducted in the rural areas of Sardinia, e.g., [33,34], education (*χ*^2^ = 32.81, df = 1, *p* < 0.0001) was not counterbalanced across the participants. Similarly, in the current study, gender was not equally distributed in the sample (*χ*^2^ = 6.48, df = 1, *p* = 0.011). Table 1 summarizes the characteristics of the sample.

### 2.2. Materials

Each participant completed the following battery of tasks:

The preliminary interview by Fastame and Penna [35] was administered to collect socio-demographic (e.g., gender, marital status) and lifestyle (e.g., smoking, hours per week spent for outdoor leisure activities or gardening) information of the respondents. Following previous research [22], years of education, the number of hours spent every week for leisure activities (i.e., calculated in terms of total hours spent for gardening and outdoor socio-culturally oriented activities) were used as proxies for cognitive reserve.

The Mini-Mental State Examination (MMSE) [36] is a 20-item screening test assessing general cognitive efficiency. Scores were adjusted for age and years of education [37]. The maximum score is 30 and a score <24 was used to detect participants with suspected cognitive impairment.

The Perceived Physical Health index (PHYS) [38] was used for the self-assessment of physical health by using a single item on a Likert scale from 0 (very bad) to 10 (excellent).

The Cognitive Failure Questionnaire (CFQ) [39], Italian version: [40] was used as a metacognitive measure of the occurrence of the motor, perceptual, and memory errors. This tool encompasses 25 items describing some daily life situations (e.g., “do you forget the name of people”). For each statement, the respondent had to assess the occurrence of that situation during the past 6 months on a Likert scale ranging from 0 (i.e., never) to 4 (i.e., very often). The total score was computed summing the given responses. According to the national norms, a score ≥22.5 indicated a general liability to cognitive failures. The internal consistency of this tool is expressed by a Cronbach’s alpha of 0.85 [40].

The Scale of Positive and Negative Experience (SPANE) [41], Italian version: [42] was used for the assessment of hedonic well-being, since the tool encompasses 6 items relative to positive experiences (e.g., during the past 4 weeks I felt joyful) and 6 items concerning negative feelings (e.g., during the past 4 weeks I felt sad). Each respondent self-rated the occurrence of each described affective experience during the past 4 weeks on a Likert scale ranging from 1 (i.e., very rarely or never) to 5 (i.e., very often or always). The total score was calculated subtracting the sum of the negative subscale (i.e., ranging from 6 to 30) from the total score of the positive items (i.e., ranging from 6 to 30). Therefore, the total score could vary from −24 (i.e., unhappiest possible) to 24 (i.e., highest affect balance). The internal consistency of this questionnaire ranges from 0.91 for the Positive Subscale to 0.92 for the Negative Subscale [42].

The Flourishing Scale [41] was administered as a self-report measure of eudaimonic well-being. This questionnaire encompasses 8 items designed for the assessment of wellness related to social relationships (e.g., my social relationships are supportive and rewarding), an active life engagement (e.g., I am engaged and interested in my daily activities), and purposeful and meaningful life (e.g., I lead a purposeful and meaningful life). For each statement, the participants had to rate their degree of agreement on a Likert scale ranging from 1 (Complete agreement) to 7 (Complete disagreement). The range total score is included between 8 and 56. This questionnaire presents adequate internal consistency, since Cronbach’s alpha is 0.88 [42].

The Vocabulary Subtest of the Wechsler Adult Intelligence Scale (WAIS) [43], Italian version: [44] that was used to measure vocabulary knowledge (e.g., what does ‘bed’ mean?), is considered a measure of crystalized verbal intelligence, and it was used as a cognitive reserve proxy. Participants had to provide the definition (i.e., lexical retrieval from semantic memory) of 35 words having high or low frequency of use in Italian. Each definition was assessed using the criteria provided in the manual of the test, assigning 0–2 scores to each stimulus, according to its correctness and completeness. The maximum possible score is 70.

Raven’s Colored Progressive Matrices (CPM) [45], Italian validation: [46] was administered to assess abstract and logical non-verbal reasoning skills by solving 36 visuo-spatial problems. Participants were asked to select among six alternatives the stimulus necessary to complete a geometrical pattern or a series. The performance was assessed in terms of the total number of correct responses (maximum total score = 36).

The Number Comprehension battery [47] includes three tasks assessing the capacity to compare numerosity (e.g., blue squares) showed simultaneously in two panels, to mark a given number on a horizontal line defined by its extremes (i.e., number line task), and to point to the right number among some alternatives (i.e., digit comparison task), respectively. The performance was rated as the total number of correct responses (maximum score = 19). The internal consistency of this tool is expressed by a Cronbach’s alpha ranging from 0.71 for the digit comparison task, to 0.73 for the number line task, and 0.8 for the numerosity comparison one [47].

The Mental Calculation battery [47] encompasses three tasks assessing simple mental calculation skills (i.e., one-digit additions, subtractions, and multiplications, respectively). For each participant, the total number of correct responses was computed (maximum score = 18). Cronbach’s alpha ranges from 0.5 for the mental subtractions (e.g., 13 − 4), to 0.61 for the mental multiplications (e.g., 9 × 6), and 0.7 for the mental additions (e.g., 5 + 7) [47].

### 2.3. Procedure

Each participant was individually tested in a quiet room of his/her own home in two separate sessions conducted within one week. First, the MMSE was administered, and if the score was ≥24, the socio-demographic interview was proposed. Then, the presentation order of the Cognitive Failure Questionnaire, SPANE, Flourishing, and Vocabulary was counterbalanced across the participants, according to the Latin Square procedure. During the second session, the presentation order of the remaining cognitive measures was also counterbalanced. To reduce the fatigue effect, the second author read aloud each item of the questionnaire/test and wrote the responses on the response sheet. Every time it was requested, a pause was given between the administration of two consecutive tasks. The experimental sessions lasted approximately 90 min in total.

### 2.4. Ethical Approval

The study was conducted in accordance with the ethics standards of the institutional research committee and with the 1964 Helsinki Declaration and its later amendments. Ethical approval for this study was obtained on 20th July 2018 from the ethical committee of the Department of Pedagogy, Psychology, Philosophy of the University of Cagliari. Written informed consent was given by all participants prior to participation.

## 3. Results

First, Pearson’s product-moment coefficients were calculated between cognitive reserve (i.e., assessed in terms of time spent for leisure activities, years of education, and vocabulary knowledge), global cognitive efficiency (MMSE), subjective physical health (i.e., PHY), numeracy skills (i.e., mental calculation and number comprehension), cognitive failures (i.e., CFQ), and hedonic (i.e., SPANE) and eudaimonic (i.e., Flourishing Scale) well-being indexes, respectively. These analyses were performed to check for multicollinearity and to examine the associations among the above-mentioned variables. The outcomes are illustrated in Table 2.

Based on the results of the correlational analyses, two regression analyses using the stepwise method were performed to explore whether number comprehension, time spent for leisure activities (i.e., Time), subjective physical health (i.e., PHY), and cognitive failures measure (i.e., CFQ) predicted hedonic well-being index (i.e., SPANE), and whether years of education, time spent for leisure activities, metacognitive efficiency (i.e., CFQ), and perceived physical health (i.e., PHYS) accounted for the eudaimonic well-being score (i.e., Flourishing), respectively. It was found that approximately 23% of the variance in the SPANE condition [R^2^ = 0.25, adjusted R^2^ = 0.233, F (4,144) = 12.25, *p* < 0.0001] was predicted by CFQ (b = −0.185, β = −31.5, t = −4.17, *p* < 0.0001), time spent for leisure activities (b = 0.082, β = 18.6, t = 2.51, *p* = 0.013), number comprehension (b = 1.291, β = 0.194, t = 2.65, *p* = 0.009), and PHYS (b = 0.831, β = 0.194, t = 2.58, *p* = 0.011). Additionally, 15% of the variance relative to the Flourishing scale index [R^2^ = 0.173, adjusted R^2^ = 0.151, F (4,148) = 7.62, *p* < 0.0001] was explained by CFQ (b = −0.075, β = −0.196, t = −2.53, *p* = 0.012), time (b = 0.062, β = 0.22, t = 2.90, *p* = 0.004), years of education (b = −0.193, β = −0.158, t = −2.12, *p* = 0.036), and PHYS (b = 0.423, β = 0.153, t = 1.98, *p* = 0.05).

**Figure 1:**
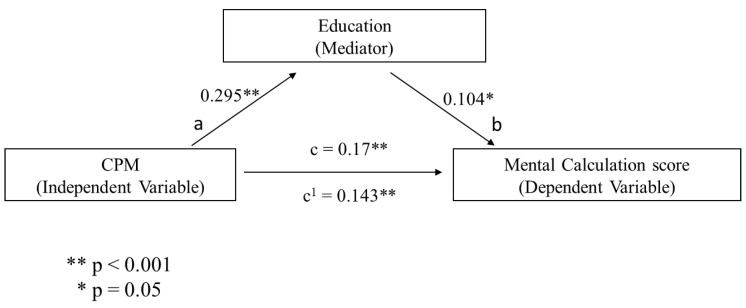
The predicted mediational pattern for Mental Calculation using CPM as the independent variable and educational attainment as mediator.

**Figure 2:**
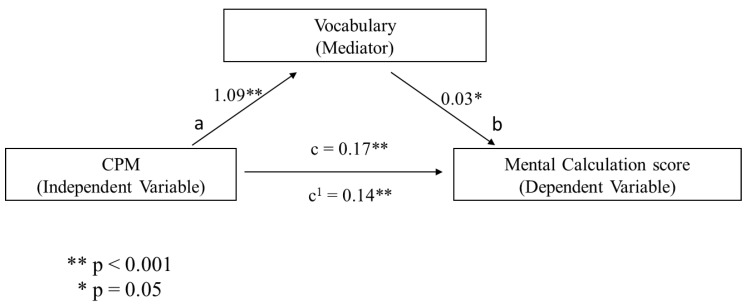
The predicted mediational pattern for Mental Calculation using CPM as the independent variable and vocabulary knowledge as mediator.

**Figure 3:**
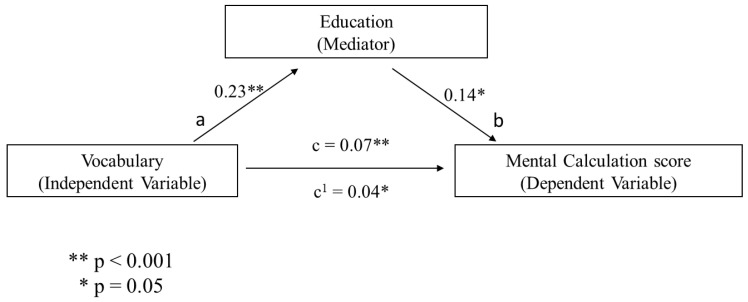
The predicted mediational patterns for Mental Calculation using vocabulary knowledge as the independent variable and educational attainment as mediator.

Additionally, a series of mediational analyses were conducted to investigate whether the association between non-verbal reasoning (i.e., assessed through Raven’s Colored Matrices) and numeracy skills (i.e., number comprehension and mental calculation, respectively) were mediated by a cognitive reserve index (i.e., assessed in terms of years of education and vocabulary knowledge, respectively) and whether cognitive reserve proxies could be used as an independent variable (i.e., vocabulary) and mediator (i.e., years of education) of the numeracy scores. Following previous evidence [48], in each mediational analysis, a bootstrap estimation approach with 5000 samples was used to test the indirect effect. For brevity, only the significant mediational models will be presented. It was found that CPM was a significant predictor of years of educational attainment (b = 0.295, SE = 0.04, *p* < 0.001, see path a in Figure 1) and years of education significantly predicted Mental Calculation (b = 0.104, SE = 0.04, *p* = 0.005, see path b in Figure 1). The mediational hypothesis was supported. After controlling for years of education (path c’ in Figure 1), CPM remained a significant predictor of Mental Calculation (b = 0.143, SE = 0.034, *p* < 0.001). Besides, the indirect coefficient was significant (b = 0.030, SE = 0.010, 95% CI = 0.001, 0.05). The standardized effect size revealed that about 18% of the variance in the Mental Calculation condition was accounted for by CPM and years of education. When the same analysis was performed using vocabulary as mediator, CPM significantly predicted vocabulary knowledge (b = 1.09, SE = 0.13, *p* < 0.001, see path a in Figure 2) and the latter significantly predicted Mental Calculation (b = 0.03, SE = 0.011, *p* = 0.02, see path b in Figure 2). Therefore, the mediational hypothesis was supported. When the impact of vocabulary knowledge was controlled for (path c’ in Figure 2), CPM remained a significant predictor of Mental Calculation (b = 0.143, SE = 0.032, *p* < 0.001). Besides, the indirect coefficient was significant (b = 0.030, SE = 0.010, 95% CI = 0.005, 0.05). Overall, the standardized effect size revealed that CPM and vocabulary knowledge predicted approximately 17% of the variance in the Mental Calculation condition. A further mediational analysis was conducted using vocabulary knowledge as an independent variable, years of education as a mediator, and Mental Calculation as a dependent variable. Vocabulary knowledge significantly predicted education (b = 0.23, SE = 0.016, *p* < 0.001, see path a in Figure 3) and years of education significantly predicted the Mental Calculation score (b =0.14, SE = 0.05, *p* = 0.005, see path b in Figure 3). These results showed that the mediational model was supported. When the impact of years of education was controlled for (path c’ in Fig. 3), vocabulary knowledge remained a significant predictor of the Mental Calculation score (b = 0.04, SE = 0.02, *p* = 0.042). Furthermore, the indirect coefficient was significant (b = 0.030, SE = 0.011, 95% CI = 0.01, 0.05). The standardized effect size documented that vocabulary knowledge and years of education predicted approximately 47% of the variance in the Mental Calculation condition. Figure 1, Figure 2 and Figure 3 illustrates these results.

Finally, two Analyses of Covariance (ANCOVAs) were carried to investigate the impact of age group (i.e., Young-Old vs. Very-Old) on number comprehension and mental calculation skills, respectively, controlling for the effects of years of education and global cognitive efficiency (i.e., MMSE). The effects of education [Number Comprehension: F(1,145) = 6.51, *p* = 0.012, ηp^2^ = 0.04; Mental Calculation: F(1,147) = 20.23, *p* < 0.001, ηp^2^ = 0.12] and MMSE [Number Comprehension: F(1,145) = 5.23, *p* = 0.024, ηp^2^ = 0.03; Mental Calculation: F(1,147) = 25.60, *p* < 0.001, ηp^2^ = 0.15] were significant in both the numeracy conditions. In contrast, the significant effect of age group was found in the Number Comprehension condition [F(1,145) = 5.45, *p* = 0.021, ηp^2^ = 0.04], whereas it approached significance in the Mental Calculation one [F(1,147) = 3.52, *p* = 0.063]. Overall, the Young-Old group performed the Number Comprehension task better (M = 17.47, SD = 1.18) than the Very-Old one (M = 16.80, SD = 1.09), whereas these significant differences were not replicated in the Mental Calculation condition (M = 17.18, SD = 1.3 for the young-Old group vs. M = 15.9, SD = 2.7 for the Very-Old group).

## 4. Discussion

The primary goal of the current investigation was to clarify the contribution of two distinct numeracy skills (i.e., number comprehension and mental calculation) in predicting hedonic and eudaimonic well-being measures in late adulthood, respectively. To our knowledge, this is the first study in which the relationship between perceived hedonic and eudaimonic well-being and number processing has been investigated in late adulthood. Thus, highlighting the exploratory nature of this investigation, for the first time the SPANE and Flourishing Scale measures were proposed to a group of Italian community-dwellers in combination with some numeracy tasks validated for the Italian older population.

Despite the paucity of studies, overall, current preliminary findings confirm and extend previous outcomes e.g., [28], suggesting at least three conclusions. First, it was found that number comprehension explains a marginal (i.e., approximately 3%) but still a significant proportion of the variance in the SPANE condition. Therefore, current findings extend previous research about the relationship between numeracy and mental health in older adults [8,26,28] and suggest that the capacity to understand and express judgments about quantities is crucial for avoiding distress and unpleasant emotions, and for promoting happiness and life satisfaction in the daily life of older people. Moreover, as expected, eudaimonic well-being was predicted by metacognition [31,32], and a cognitive reserve proxy (i.e., time spent on leisure activities) [34,38]. In short, one can conclude that being capable of number processing, trusting one’s cognitive efficiency, and assuming an active lifestyle boosting physical (i.e., gardening and sports) and mental (i.e., socially-oriented intellectual leisure activities) health seem to be crucial for the enhancement of self-reported well-being in late adulthood.

Furthermore, current findings documented that cognitive reserve assessed in terms of educational attainment and vocabulary knowledge is significantly related both to number comprehension and mental calculation in late adulthood. Besides, extending the evidence by Barbosa et al. [18], Delazer et al. [2], and Mulas et al. [21], current outcomes revealed that years of education and vocabulary knowledge mediated the relationship between non-verbal reasoning and mental calculation. Additionally, present results showed that 47% of the variance in the Mental Calculation condition was predicted by two cognitive reserve proxies, that is, the lexical knowledge (assessed through the Vocabulary task) and years of education.

Finally, in line with previous studies [2], when the effects of global cognitive efficiency and education were controlled for, younger participants outperformed the oldest ones in the Mental Calculation task, whereas no significant differences were found between the two groups in terms of number comprehension.

However, some limitations about the current investigation need to be discussed. Specifically, the paucity of participants, the limited battery of tools used in this study, and the low educational attainment of the participants suggest replicating the study using wider samples and further measures of metacognitive (e.g., questionnaires assessing current perception about memory functioning), cognitive (e.g., verbal and visuo-spatial working memory and inhibition tasks), numeracy (e.g., financial numeracy tasks), and well-being. Future research should also deeply examine the impact of cognitive impairment on number processing in late adulthood. Indeed, there is emerging evidence [14,17,25] about the implication of cognitive impairment and low numeracy skills in facilitating the financial exploitation of older individuals. This suggests that the impact of mild cognitive decline and low numeracy on financial decision making needs to be carefully examined in late adulthood.

## 5. Conclusions

This study documented the interplay among numeracy skills, perceived hedonic well-being, and some cognitive reserve proxies in late adulthood. In short, older people with higher education and better lexical knowledge perform better in mental calculation tasks, which in turn can play a crucial role in daily life decision making. Indeed, from an applied viewpoint, it is important to bear in mind that lower numeracy can negatively affect health-related and financial decision making, and can be a significant marker of adverse health and financial outcomes in late adulthood [2,4,9]. At present, financial exploitation is still an underestimated phenomenon among older people [49], but it is quite pernicious [12], especially among older individuals living alone and those cognitively impaired. Thus, considering the impact of number acuity on the daily life of older people (e.g., bank transitions, medicines intake, shopping), in order to promote health-related decision making and prevent financial exploitation [15], current outcomes suggest that screening programs should be planned to test the efficiency of numeracy skills and those specific interventions enhancing cognitive reserve (e.g., time spent for physical and occupational activities) and numeracy skills in later life—and especially mental calculation—should be implemented to promote an active engagement with life and therefore healthy behaviors (e.g., therapeutic compliance) in late adulthood [10]. Concerning this, a recent stream of research suggests that the effect of training empowering numeracy skills in older people is promising if they are tailored to the specific needs of older individuals [50]. Nonetheless, the paucity of studies conducted in late adulthood revealed that in clinical practice, the implementation of specific interventions aimed at screening and enriching numeracy (i.e., together with cognitive functions such as working memory and metacognitive competencies) of older people is still too limited and should be encouraged.

## Figures and Tables

**Table 1:** Socio-demographic information and global cognitive efficiency (i.e., Mini-Mental State Examination (MMSE)) scores collected from all the participants in the study.

Variable	Young-Old	Very-Old	*χ* ^2^	t	df	*p*
	Group	Group				
n	73	85	0.911		1	0.34
Gender			0.13		1	0.72
males	28	35				
females	45	50				
Age (years)	M = 70.7	M = 80.1				
	(SD = 2.5)	(SD = 4.3)				
Age range (years)	65–74	>74				
Education (years)	M = 9.5	M = 6.3		5.1	156	<0.001
	(SD = 4.2)	(SD = 3.5)				
Educational attainment			10.72		1	0.001
low	44	71				
high	29	14				
Marital status			11.27		1	0.001
Single/widow	21	47				
Married	52	38				
Living			10.56		1	0.001
alone	12	34				
with others	61	51				
Time spent on hobbies	M = 29.9	M = 22.3		2.77	156	0.006
(hours per week)	(SD = 18.4)	(SD = 16.4)				
MMSE score	M = 26.6	M = 26.09		1.77	156	0.08
	(SD = 1.3)	(SD = 2.4)				
Signs of Cognitive Decline			10.42		1	0.001
No	72	71				
Yes	1	14				

**Table 2:** Person’s correlations among numeracy (i.e., Mental Calculation, Number Comprehension), time spent for leisure activities (i.e., Time), years of education (i.e., Education), global cognitive efficiency (i.e., MMSE), perceived physical health (i.e., Perceived Physical Health index (PHYS)), metacognitive efficiency (i.e., Cognitive Failure Questionnaire (CFQ)), hedonic (i.e., Scale of Positive and Negative Experience (SPANE)) and eudaimonic (i.e., Flourishing) well-being, and non-verbal reasoning (i.e., Raven’s Colored Progressive Matrices (CPM)), respectively.

	1	2	3	4	5	6	7	8	9	10	11
**1 Mental Calculation**	—										
**2 Number Comprehension**	0.432 ***	—									
**3 Time**	0.117	0.116	—								
**4 Education**	0.437 ***	0.308 ***	0.031								
**5 MMSE**	0.096	0.152	0.151	0.211 *	—						
**6 PHYS**	0.029	−0.059	0.161 *	0.014	−0.031	—					
**7 CFQ**	0.068	0.138	−0.138	0.04	0.019	-0.237 **	—				
**8 SPANE**	0.014	0.161 *	0.283 **	−0.003	−0.034	0.276 ***	−0.365 ***	—			
**9 Flourishing**	−0.061	−0.008	0.269 **	−0.162 *	−0.177 *	0.234 **	−0.269 ***	0.48 ***	—		
**10 Vocabulary**	0.43 ***	0.397 ***	0.08	0.775 ***	0.422 **	−0.031	0.019	−0.034	−0.177 *	—	
**11 CPM**	0.553 ***	0.432 ***	0.19 *	0.484 ***	0.333 **	−0.06	0.184 *	0.106	−0.5	0.53 ***	—

Note. * *p* < 0.05, ** *p* < 0.01, *** *p* < 0.001.

## Data Availability

The data that support the findings of this study are not publicly available due to privacy or ethical restrictions.
